# Observing Etna volcano dynamics through seismic and deformation patterns

**DOI:** 10.1038/s41598-023-39639-9

**Published:** 2023-08-10

**Authors:** L. Scarfì, M. Aloisi, G. Barberi, H. Langer

**Affiliations:** Istituto Nazionale di Geofisica e Vulcanologia – Osservatorio Etneo, Catania, Italy

**Keywords:** Solid Earth sciences, Geophysics, Seismology, Tectonics, Volcanology

## Abstract

Geophysical data provide the chance to investigate a volcano’s dynamics; considerable information can especially be gleaned on the stress and strain patterns accompanying the internal processes and the effect of magma ascent on the main structures triggering earthquakes. Here, we analysed in detail the seismicity recorded over the last two decades on Etna volcano (southern Italy), focusing on earthquakes distribution and focal mechanism clustering; the ground deformation pattern affecting the volcanic edifice with the inflation and deflation phases was also examined. Analysed data were compared in order to shed light on possible relationships with the volcanic activity and to better understand the internal dynamics of the volcano over time. Significant steps during or shortly before major eruptions in the seismic strain release and ground deformation temporal series highlight a straightforward relationship between seismicity occurring at shallow level, inflation/deflation and volcanism. Furthermore, at depths greater than 5–7 km, down to about 20 km, the orientation of the P- and T-axes clearly indicate the existence of a pressure source in the central part of the volcano. All the results underline that the stress field related to the volcano plumbing system interferes with the regional field, partly overriding it.

## Introduction

Volcanic phenomena can be understood as the expression of magma dynamics beneath volcanoes. Geophysical data, such as ground deformation, the distribution of seismicity and the analysis of the local stress field, provide important information on the processes ongoing at depth.

On active volcanoes like Mt. Etna, changes observed in geophysical data are often related to variations of the physical state of the volcano. Indeed, the pressurization inside magmatic bodies and their intrusions into the shallow crust influence the stress field, promoting fault slip and movements of unstable volcano sectors (e.g. Refs.^1–3^). Changes of the local stress field in space and time and in the seismicity have been reported at many volcanoes (e.g. Refs.^4–7^), while continuously recorded ground deformation data have been related to the processes of magma migration in the upper levels of the plumbing system (e.g. Refs.^8–11^). Therefore, the analysis of the seismic and ground deformation patterns can provide insights on the state of a volcano and on the magma transfer within the crust^12–14^. It contributes to understanding the volcanic hazard and the design of mitigation strategies.

**Figure 1:**
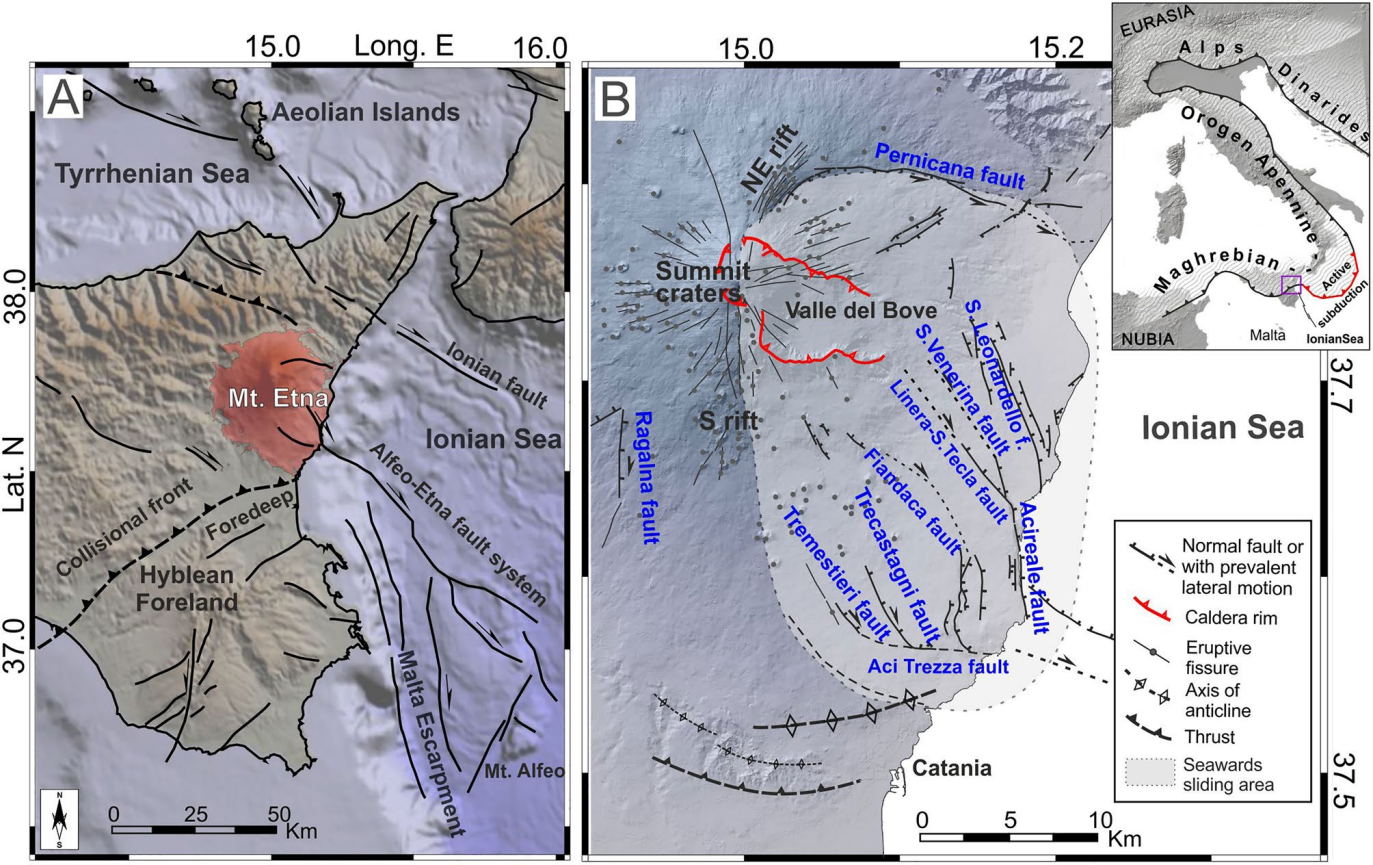
(**A**) Tectonic sketch-map of eastern Sicily and the Ionian offshore. (**B**) Main structural elements of Mt. Etna (based on Ref.^17^). Inset shows the Alpine-Apenninic-Maghrebian orogen in the context of the Eurasia-Nubia convergence. Topography is from Ref.^60^. The maps were created using GlobalMapper (version 24.0.2; https://globalmapper.it/) and Generic Mapping Tools (version 6.0; https://www.generic-mapping-tools.org/).

Mt. Etna is a 3300 m high Quaternary basaltic stratovolcano, formed in the geodynamic setting of the convergence between the African and European plates; it is located in the seismically active region of the eastern coast of Sicily (in central Mediterranean), at the south-western edge of the Ionian/Calabrian subduction zone and at the front of the Apennine Maghrebian fold and thrust collisional belt^15,16^ (see Fig. 1). The volcanic edifice is intersected by a number of outcropping faults, some of which are linked to major regional structural systems^17^. These faults often generate shallow earthquakes which may cause severe damage despite their relatively small magnitudes. Thus, kinematics, stress and strain fields characterizing Mt. Etna are topics addressed in numerous papers (e.g. Refs.^4,18–22^). Nevertheless, most of these studies refer to limited areas and specific periods of time (e.g. eruptive events); a long-term multidisciplinary analysis, helping to better define more general models for the sources governing seismic activity and volcanism, is quite rare (e.g. Ref.^23^). Moreover, there are considerable difficulties in distinguishing seismicity related to the volcanic processes (phases of inflation or deflation of the volcano due to volume variations in the magma storage, or to dike intrusions) from the seismicity linked to the regional dynamics.

In this study, we have analysed various aspects of seismicity of Etna volcano. We considered the patterns inferred from earthquake locations, focal mechanisms and cumulative seismic strain release, with the aim of shedding light on relationships between the seismological picture, the sources of ground deformation and the volcanic activity. We seek to provide a comprehensive interpretation of the dynamics of the volcano in the framework of seismotectonic considerations, taking into account ground deformation and seismic data collected over the entire Mt. Etna area during the last two decades. We investigated the question whether the evolution of the magmatic system as revealed by the ground deformation modelling corresponds to the spatial changes in the seismic activity and local stress field inferred from the focal mechanisms.

### Etna volcano activity

In the last 20 years, the nearly continuous volcanic activity of Etna has been particularly intense, with several flank or summit eruptions, periods of continuous Strombolian activity and hundreds of explosive events generating lava fountains (for a review of the Etna activity^24–26^). In Table 1, we compiled a scheme with the major eruptive events and the inflation periods occurring from 2002 until the time of this study (2021); the selected events showed clear evidence of ground deformation and allowed a modelling^11^.

**Table 1:** Main volcanic activity observed at Mount Etna from 2002 to 2021.

Starting	Ending	Volcanic activity
01/01/2002	26/10/2002	Inflation
27/10/2002	28/01/2003	2002 flank explosive eruption
29/01/2003	07/09/2004	Inflation
08/09/2004	08/03/2005	2004 flank effusive eruption
09/03/2005	13/07/2006	Inflation
14/07/2006	15/12/2006	2006 flank effusive eruption
16/12/2006	12/05/2008	Inflation
13/05/2008	07/09/2009	2008 explosive/effusive eruption
02/08/2008	31/12/2010	Inflation
31/12/2010	20/05/2011	Stasis
20/05/2011	16/07/2011	Inflation
16/07/2011	17/10/2011	14 lava fountains
17/10/2011	26/04/2012	7 lava fountains
26/04/2012	15/02/2013	Inflation
15/02/2013	30/04/2013	13 lava fountain
01/05/2013	23/10/2013	Inflation
21/01/2014	01/04/2014	Effusive eruption from summit craters
05/07/2014	15/08/2014	Effusive eruption from summit craters
15/08/2014	01/12/2015	Inflation
02/12/2015	06/12/2015	4 lava fountains
07/12/2015	16/05/2016	Inflation
17/05/2016	26/05/2016	3 lava fountains
27/05/2016	23/12/2018	Inflation
24/12/2018	26/12/2018	2018 eruption
27/12/2018	15/02/2021	Inflation
16/02/2021	31/12/2021	58 lava fountains

The seismicity in this time span was characterized both by sporadic activity as well as seismic swarms, only partly linkable to the volcanic activity (as in the case of the very shallow syn-eruptive seismicity).

### Ground deformation

**Figure 2:**
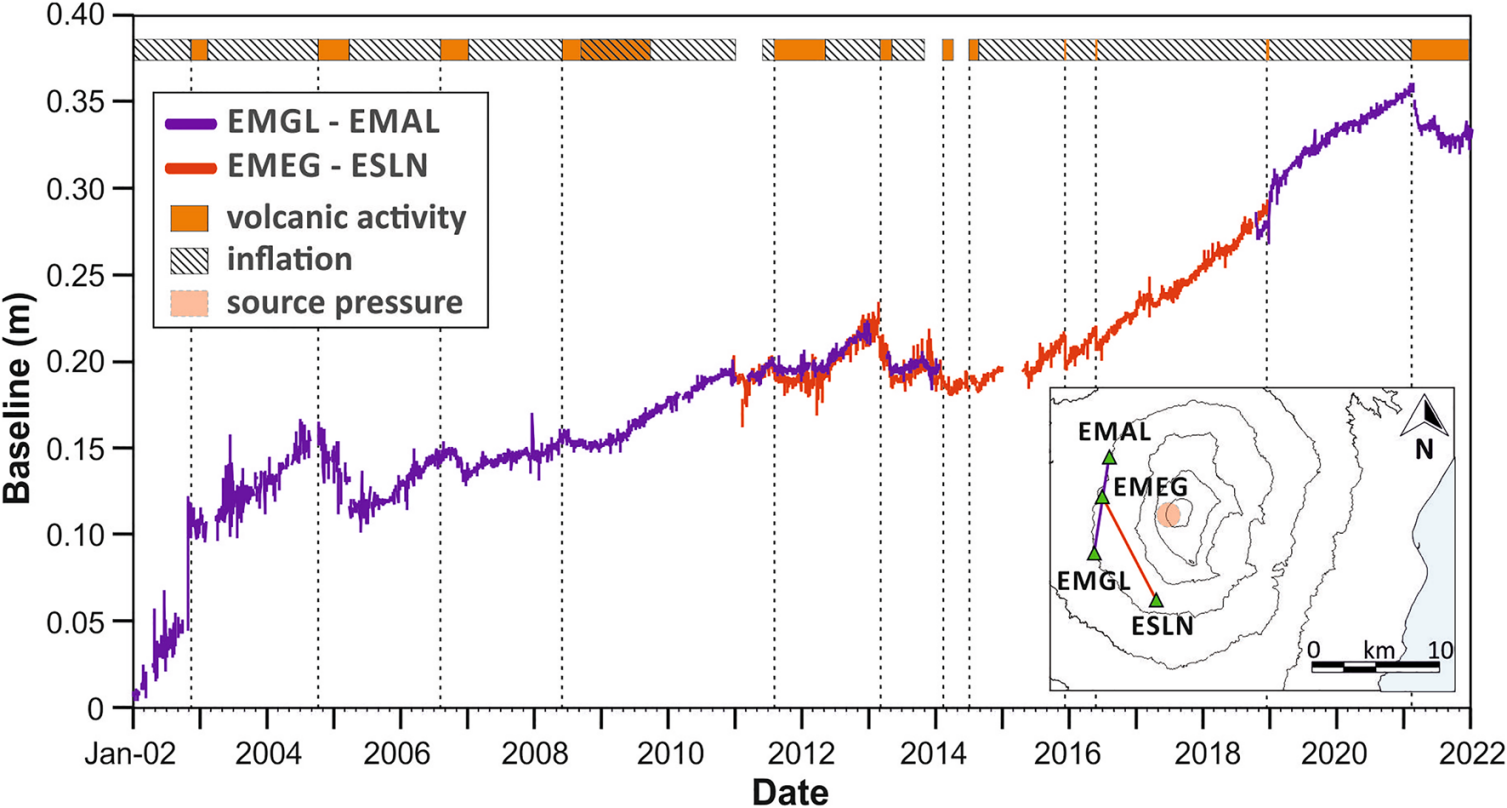
Time‐series (2002–2021) of the daily distance variations between two pairs of benchmarks (green triangles in the inset) located on the western flank of Mt. Etna. In the inset, the map showing the location of the two GPS baselines analysed here and the typical location of the inflation sources found during the studied time period^30^ (red circle). The bar at the top indicates the ongoing volcanic activity, distinguishing between periods of exclusive inflation, characterized by the absence of an intense lava outpouring, and periods of explosive and/or effusive processes. Dotted lines highlight the beginning of main volcano activities.

The improvement of the ground deformation monitoring system from 2001 on (see Ref.^27^ and Fig. S1 in Supplementary Information) has allowed to perform detailed modelling of acting pressure sources. Many researchers (e.g. Refs.^27–30^) clearly delineated a pathway of the magma rising towards the surface, which borders the western side of the high seismic velocity body underneath the summit craters, visible in several tomographic studies (e.g. Ref.^31^). Along this pathway, the magma can accumulate and differentiate at various levels. In particular, Aloisi et al.^30^ proposed the presence of inflation sources located in a volume at a depth between about 5 and 8 km b.s.l (see inset in Fig. 2).

Regarding the ground deformation pattern affecting the volcanic edifice, we focus on distance measurements between benchmarks located on the western flank of the volcano, in order to identify the inflation/deflation phases. Part of the data is already available in the literature (see Ref.^16^); in addition, the data since 2019 until the end of the year 2021, were analysed using the software GipsyX/RTGx^32^. We prefer the baselines on the western flank to the ones deployed on the eastern side as the former are much less affected by major faulting and eruptive fracturing (see e.g. Ref.^29^). Indeed, the eastern and southern flanks of the volcano, crossed by NNW-SSE fault systems (Fig. 1B), represent an unstable sector of the volcano, characterised by aseismic and seismic deformations accompanied by seaward motions (tectonic and gravity‐driven^22,23,33^). Thus, the behaviour of the western flank is typically considered a clear indicator of the volcano state related to the processes ongoing in the plumbing system (e.g. Refs.^34,35^). By default, we focused on the baseline EMGL-EMAL; in addition, also the baseline EMEG-ESLN was considered (see inset in Fig. 2). Their orientation differs little but both baselines are concentric with respect to the typical location of the inflation sources (Fig. 2); this means that they are similarly stressed by the source and their trend is almost identical, as we verified during times when both baselines could be measured. We therefore used EMEG-ESLN when EMLG-EMAL was unavailable.

The ground deformation curve (Fig. 2) shows a general trend of inflation over the 20 years. On occasion of major eruptions, this trend is interrupted by rather short-lived periods of deflation; for instance, we note about 1 year of deflation in 2005 and also from 2013 to 2015 (see Ref.^30^). Rapid inflation occurred from 2002, during the strong 2002/03 flank eruption, until the 2004 eruption^36^. Inflation accelerated from 2015 until 2021. This phase accounts for almost 50% of the inflation encountered in the years 2002–2021. In 2021, we witnessed a major deflation, linked to the paroxysmal activity with about 60 lava fountains^27^.

### Seismicity: overall picture

**Figure 3:**
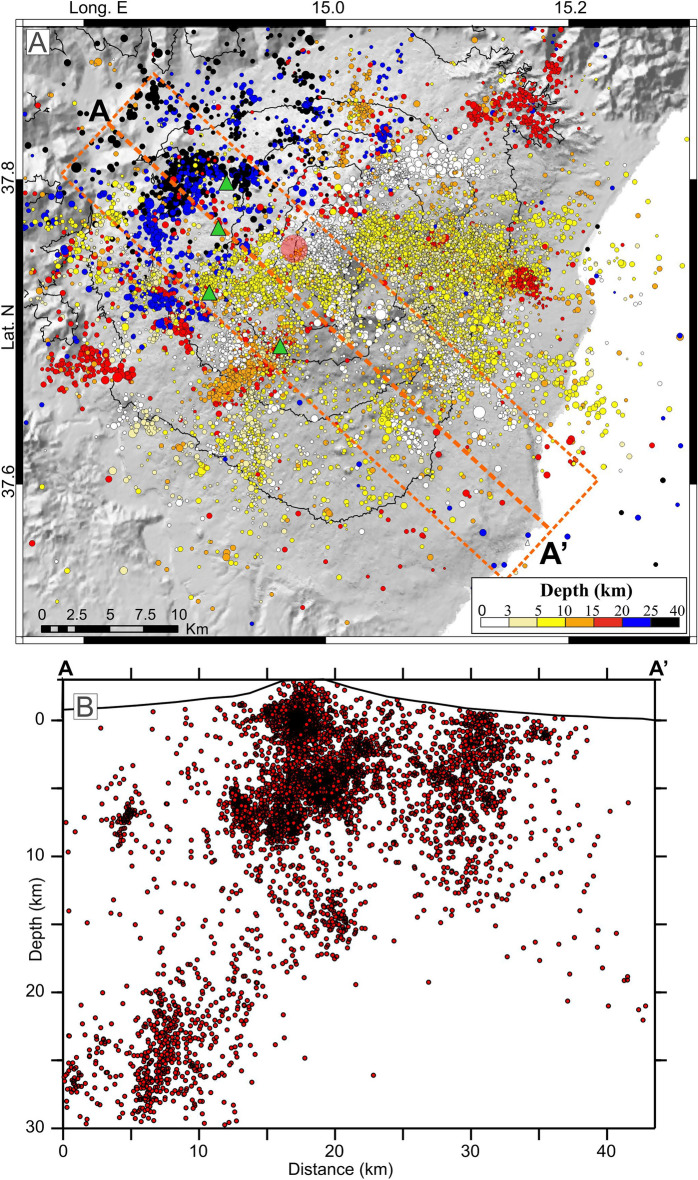
Location in map view (**A**) and cross-section (**B**) of the earthquakes recorded in the Mt. Etna area in the period 2002–2021. The orange dashed line in the map indicates the trace and width of the section; the green triangles and the red circle in the central area of the map represent the GPS stations and the location of the inflation source respectively, from Fig. 2. Topography is from Ref.^60^. The maps were created using QGIS Desktop (version 3.16.1; https://www.qgis.org/it/site/) and Generic Mapping Tools (version 6.0; https://www.generic-mapping-tools.org/).

Over the period 2002–2021, about 19,000 events (brittle-shear failure type), with magnitude mostly between 1 and 2.5 (Mmax = 4.8), were recorded and located in the area of Mt. Etna, by the dense seismic network deployed on the volcano (Fig. S1 in Supplementary Information). In order to improve the picture deriving from the seismic dataset, the initial parameters (arrival times and locations, taken from Refs.^37–41^) were processed using the tomoDDPS software^42^. Compared to standard location techniques, tomoDDPS improves the accuracy of hypocentre location through a combination of absolute and differential arrival-time readings between couples of closed-spaced earthquakes. Besides, the code allows computing the seismic ray-tracing in a 3D velocity model; here we used a model derived by the integration of passive and active seismic data^43^. The final locations (Fig. 3) resulted with an average uncertainty of 0.10 ± 0.04 km in both horizontal and vertical coordinates, and an average root-mean-square travel-time residual of 0.02 ± 0.005 s.

The seismicity on Etna is rather heterogeneously distributed. As a general trend, hypocentre depth increases from E, ESE towards NW. Shallow foci, with a depth of less than 5 km, are frequent in the central part of the volcano, as well as on its eastern and southern flanks. A similar distribution is found for the earthquakes with depths of about 5–10 km. Foci with depth between 10 and 15 km are frequent in the SW part of the area, but also to the N and NNE of the summit craters, while deeper events (depth in the range of 20–35 km), are located in the NW part of the volcanic edifice. A fairly clear boundary between domains with shallow and deeper foci can be outlined; it crosses the volcano from SW to NE, intersecting the summit area.

The distribution of the hypocentres reflects the interaction of local effects due to the dynamics of the volcano, and the regional tectonic framework of the area. In this picture, the large amount of seismicity in the central area points to the mid-shallow plumbing system, while the progressive increase of focal depth towards NW can be understood as an expression of the crustal convergence that takes place beneath the thin volcanic cover; i.e., under the volcano we encounter the NW subduction of the Hyblaean foreland crust below the fold and the thrust system of the SE verging Sicilian chain (see Refs.^16,44,45^). Likewise, the large number of hypocentres of the eastern sector, mirrors the activation of the numerous faults characterising that flank, believed to be linked to a regional transtensional fault system extending offshore^17,33^.

### Seismogenic volumes and strain rate

We quantified the seismic activity over time in calculating the cumulative curves of seismic strain within some key volumes of the volcanic area. The seismic strain curves were obtained using Richter’s relation^46^ for seismic energy (E in erg), i.e., log(E) = 9.9 + 1.9M_L_ − 0.024M_L_^2^.

For the definition of the key volumes, we started from the depth distribution of the foci. By ordering the events according to their depth (Fig. S2A in Supplementary Information), we noticed changes in the distribution curve around 3 km, 7–8 km and 17–18 km; these limits are appropriate even if the seismic energy release at the various depths is considered (Fig. S2B in Supplementary Information). We therefore decided to distinguish four main depth ranges, i.e. < 3 km, 3–7 km, 7–17 km and > 17 km.

**Figure 4:**
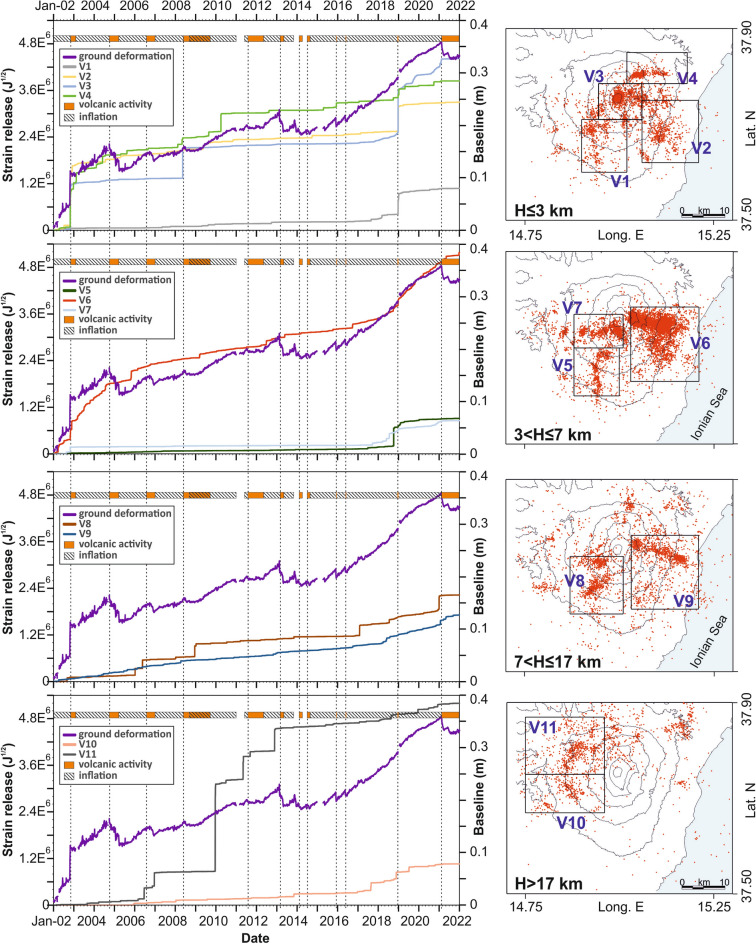
Curves of the cumulative seismic strain release, calculated within key crustal volumes (V1–V11; see maps on the right), and their comparison with the ground deformation curve (purple). The deformation curve is estimated using a weekly mean. Seismic events (red dots) and the related strain release curves are separated in depth levels, indicated on the bottom left of the maps (see text for more details). The bar at the top of the panels and the dotted lines are the same as in Fig. 2.

To identify the horizontal boundaries of the key volumes, we mapped the seismic energy released in the volcanic area. Specifically, we defined a three-dimensional grid, with a horizontal step of about 1 km and vertical spacing corresponding to the depth limits defined above. We calculated the sum of the energy released by the earthquakes located within the grid cells. Focusing on zones with a high release of energy, we finally identified 11 volumes, which encompass most of the seismicity of the volcano (Fig. S3 in Supplementary Information). For each, we calculated the seismic strain release curves and compared them to the ground deformation curve mentioned earlier (Fig. 4).

In the shallowest layer we distinguish four key volumes; they show a major release of seismic strain during the flank eruptions characterized by intense seismic swarms, i.e. the eruptions of 2002/03 and 2018. This is especially true for the V2 curve which refers to events localized in the eastern sector, while the curve V3, encompassing the summit craters area, shows a strong increase even during the 2008 eruption. V4, which refers to the NE Rift and the Pernicana fault sector, i.e. the northern edge of the volcanic block subject to eastward sliding, reveals a certain degree of activity even in non-eruptive periods.

In the layer between 3 and 7 km, we have distinguished 3 key volumes. Volume V5 encompasses foci in the SW part of the area, V6 lies on the eastern flank of the volcano, while V7 includes the summit craters and adjacent areas to the west. The similarity of seismic strain release and ground deformation curves is strongest for the volume V6, i.e. the events on the eastern flank. Moderate increments of the V7 are detected before the eruptions of 2002/03 and 2018. On the other hand, seismic strain release of V5 is strongly affected only by the December 2018 eruption.

In the layer between 7 and 17 km we consider the volumes V8 with foci located SW of the summit, and V9, which is situated E and SE of the central craters. While the seismic strain release in V9 is rather smooth, several steps can be observed in the curve for V8 which, however, do not show a particular link to the volcanic activity.

In the basement with depth larger than 17 km, we identified two volumes, V10 situated SW and V11 NW of the summit area. V11 is among the seismically most active volumes. Its curve shows marked steps just before some eruptive activities (i.e. 2006, 2011 and 2013), however the same happens in inter-eruptive periods; therefore, a clear relation to the ground deformation curve or volcanic activity is lacking. V10 shows a lower degree of seismic activity, again poorly linked to ground deformation or volcanic activity. However, a common feature of several curves is a rate increase before the 2018 eruption.

With the aim of evaluating how comparable the seismic and deformation trends were, we defined a measure of similarity between the normalized curves of ground deformation and strain release for the 11 key volumes. We calculated the sum of the absolute differences between the normalized curves. Formally, it is obtained for each of the volumes, i.e.

$$\Delta = 1/nsamp{\int }_{t=01Jan2002}^{t=31Dec2021}|\left({V}_{i}\left(t\right)-D\left(t\right)\right)|dt$$

where ${V}_{i}\left(t\right)$ is the cumulative strain of the *i*-th volume and $D\left(t\right)$ the cumulative ground deformation, *t* is the time variable and $nsamp$ the number of samples (7305 in 20 years). Then, we considered the relative amount of not coinciding areas with reference to the area covered by the ground deformation curve D(t). Formally this is obtained as

$${\Delta }^{,}=\Delta /{\int }_{t=01Jan2002}^{t=31Dec2021}D(t)dt$$

(see also Supplementary Information). It should be small when the two curves are similar—such as for the pair deformation/seismic strain V6—or higher for notably differing curves, such as can be seen for the pair deformation/seismic strain V5. Taking the accumulated divergences ${\Delta }^{,}$ for each of the 11 key volumes, we obtain the values reported in Table 2.

**Table 2:** Similarity between the ground deformation and strain release curves for 11 key volumes.

Volumes	V1	V2	V3	V4	V5	**V6**	V7	V8	V9	V10	V11
${\Delta }^{,}$(%)*	54	28	13	31	60	**9**	39	21	25	48	43

*Relative amount of not coinciding areas with reference to the area covered by ground deformation curve. In bold, the value identifying the curve with the highest similarity.

From this criterion the ground deformation is most similar to the seismic strain release of V6, i.e. the volume to the east, situated at depth between 3 and 7 km. The second most similar one is the volume 3, which encompasses the shallow level of the summit area. Higher similarity might suggest that seismic activity and deformation are related.

### Focal mechanism clustering and stress orientation

Focal solutions provide useful information on the orientation and type of the rupture and the forces acting around the seismic source. In a geologically complex area, however, the differentiation in groups of “Normal”, “Reverse”, “Horizontal Strike-Slip” or “combined type” mechanisms may not be sufficient to delineate homogeneous zones of seismicity. Indeed, this classification does not include any information about the strike of the P- and T-axes; this can be limiting, for example in studies such as this one where we investigate the orientation of the stress field controlling the tectonic processes of an area.

Following a strategy proposed by Scarfì et al.^47^ (see also Ref.^48^), we used unsupervised pattern recognition. It is based on an a-priori defined metric of similarity between patterns rather than exploiting a-priori defined target information furnished by an expert (see Supplementary Information for more details). We applied these unsupervised learning techniques to moment tensors derived from fault plane solutions of Mt. Etna earthquakes. To this end, we collected basic data (locations, travel-times and first polarities) of earthquakes with focal solution from available datasets and published studies^18,19,45,49–52^ and processed them by calculating the ray-tracing in a 3D velocity model^43^. Finally, by using the FPFIT software^53^, we obtained 577 focal solutions of events with magnitude ranging from 1.3 to 4.8, recorded in the time span 2002–2021; the average uncertainty on the orientation of the nodal planes is about 10°. These focal mechanisms can be considered representative of the kinematics characterizing Mt. Etna, since they are distributed in all the main seismogenically active areas of the region.

In order to divide the focal mechanisms into homogeneous groups, we used an updated version of the software KKAnalysis^54,55^, which offers different types of unsupervised classifiers. As a first step, the dataset was processed by applying the so-called Kohonen Maps, also known as “Self-Organizing Maps” (SOM, see Supplementary Information). SOM enable reducing the dimensionality of the feature vectors, allowing an efficient representation of pattern characteristics in space and time. Similar to Cesca et al.^48^, we adopted moment tensor components as features. They can be handled with centroid-based clustering, allowing the definition of prototypes, i.e., objects which represent a number of patterns with reasonable fidelity. The algorithm grouped all the 577 focal solutions in 114 nodes, each of which can be understood as micro-clusters, whose centroid vectors form prototypes. We can substitute the original feature vectors by the prototypes which means a smoothing of the data; however, we are still dealing with a large number of groups (i.e. 114). Thus, in a second step, we performed the K-means clustering by exploiting the results obtained from the SOM. Specifically, the original moment tensors of the dataset were replaced by the ones of the prototypes, being representative of the original items. This significantly reduced the scatter of the initial data; indeed, instead of having 577 different moment tensors the variability drops to only 114 versions of feature vectors. For K-means, we can use simple rules for establishing the adequate number of clusters, namely the “Davis-Bouldin” index (see Supplementary Information). Using this index 8 clusters are preferred and we deal with a still smaller number of representative feature vectors—i.e., eight instead of the 114 SOM-nodes or the 577 original ones. The overall characteristics of each group were summarized by identifying the prototypes, i.e. patterns that are similar to all members of a cluster. This was done using the so-called Kagan angles^56^. The metric is based on the angle by which a system P, T, B must be rotated so that the axes fall in the same position as P’, T’, B’ (see Supplementary Information). The best representation is found by taking the cluster members one by one and calculating the sum of Kagan angles with respect to all other members. The representative sample is the one with the lowest sum of Kagan angles (see Ref.^47^ for further details).

**Figure 5:**
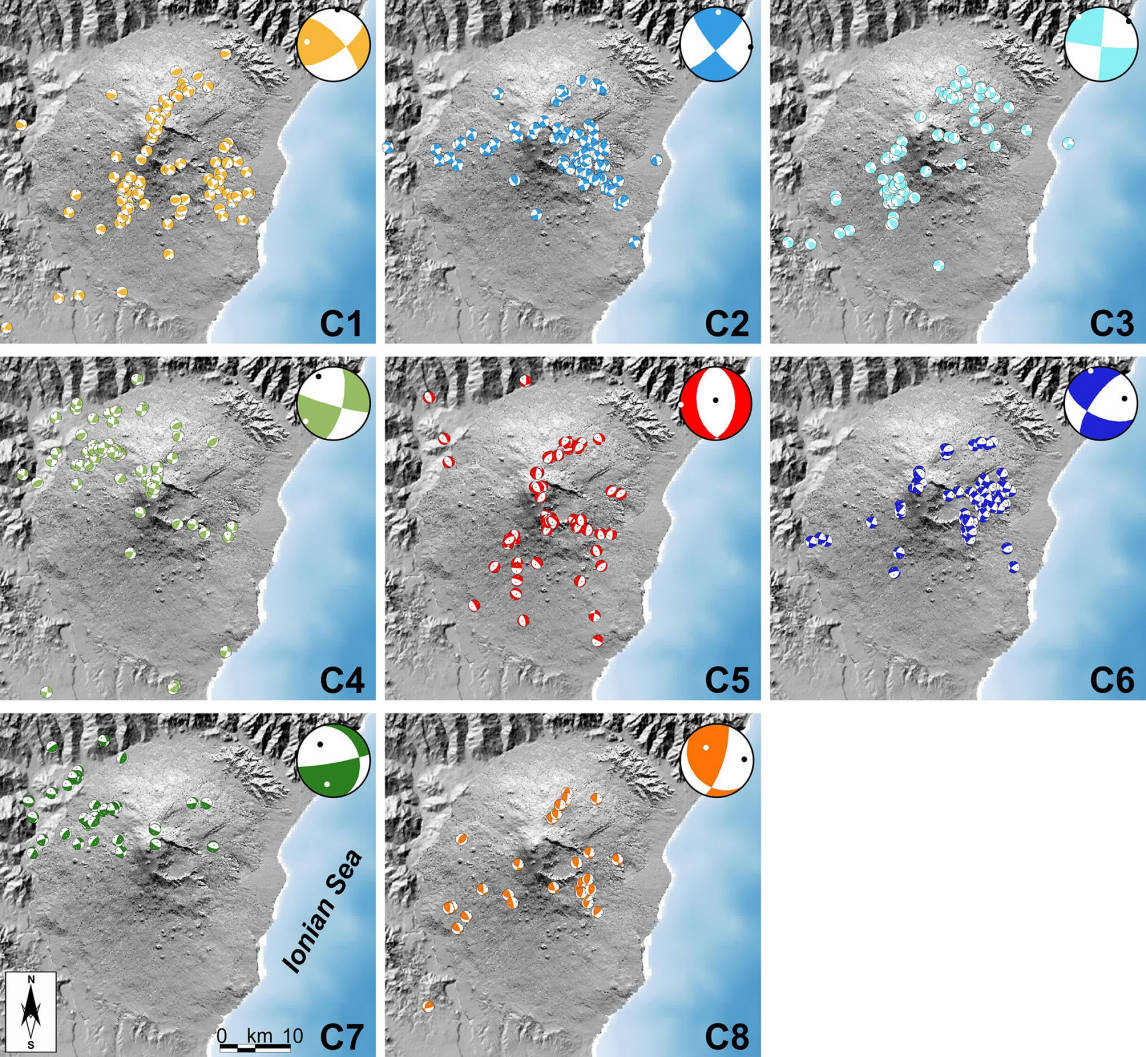
Groups of focal mechanisms derived from the clustering procedure (C1–C8). In the top right corner of the panels, the identified prototypes of each group are reported. Topography is from Ref.^60^. The maps were created using QGIS Desktop (version 3.16.1; https://www.qgis.org/it/site/) and Generic Mapping Tools (version 6.0; https://www.generic-mapping-tools.org/).

In Fig. 5, we present the distribution of the focal mechanisms in the Mt. Etna area. The colouring of the beach balls represents their membership to the clusters; the prototype beach balls of the clusters are shown in the upper right corner of each panel. Clusters 1, 2, 3, 4 and 6 correspond essentially to horizontal strike slip mechanisms. They are distinguished by differences in the direction of the P-axes, which is N for members of cluster 1, NNW for cluster 4, E and NE for clusters 2, 3 and 6. Cluster 5 consists of clear normal faulting events, even though the strike directions of the P- and T-axes show a considerable scatter. Clusters 7 and 8 are mainly of reverse type; P-axes are directed NW–SE for C7, while they spread from ENE-WSW to ESE-WNW for C8.

**Figure 6:**
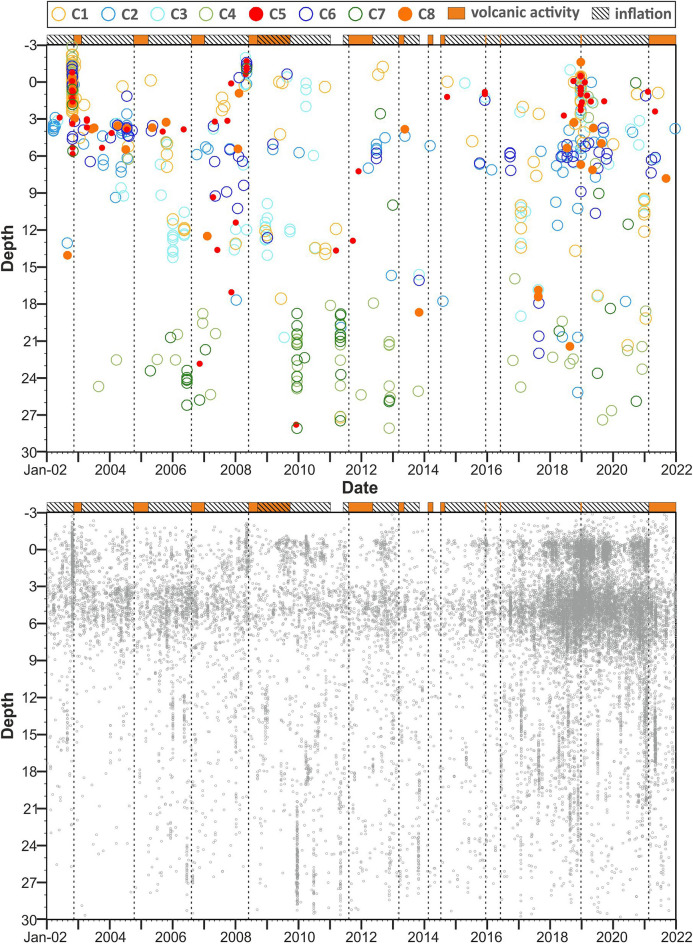
Top—distribution in time and depth of the considered focal mechanisms. Colouring is the same as in Fig. 5. As in Figs. 2 and 4, time intervals with enhanced volcanic activity are represented at the top of the figure by orange bars; cross-hatched bars indicate periods of inflation. Bottom—the distribution in depth and time of all earthquakes located in the Etna area in the period 2002–2021 is shown, for the sake of completeness.

The distribution of the clusters as a function of time and depth is shown in Fig. 6. Of note is the concentration of certain groups in distinct depth levels. In particular, the clusters C1, C5 and C8 are primarily representative of shallow events (median depth less than 4 km). They occur in the summit area or along the main fault systems of the volcano, namely the southern and north-eastern rift zones (close to the central craters) and the Pernicana fault (NE of the summit). Clusters C2 and C6 are located mainly in the central-eastern sector, in the depth range 3–7 km. The elements of the cluster C3 are located NE or SW of the summit area, at depths from very shallow to about 15 km. The foci belonging to clusters C4 and C7 are found in the NW sector, at depths over 17 km; furthermore, they tend to cluster in time, forming swarms, such as in June 2006 (before the 2006 eruption), in December 2009 or in May 2011 (before the lava fountain activity). The foci of the clusters 5 and 8 often coincide with periods where strong volcanic activity is observed. During the eruptions 2002/03 and December 2018, the two event types occurred at very similar times. This is a surprise at a first instance: cluster 5 includes mainly normal faulting mechanisms, whereas the mechanisms of cluster 8 correspond to horizontal strike slip/reverse faulting. This is probably an effect of the complex stress redistribution during the process of dike intrusion and flank movement.

**Figure 7:**
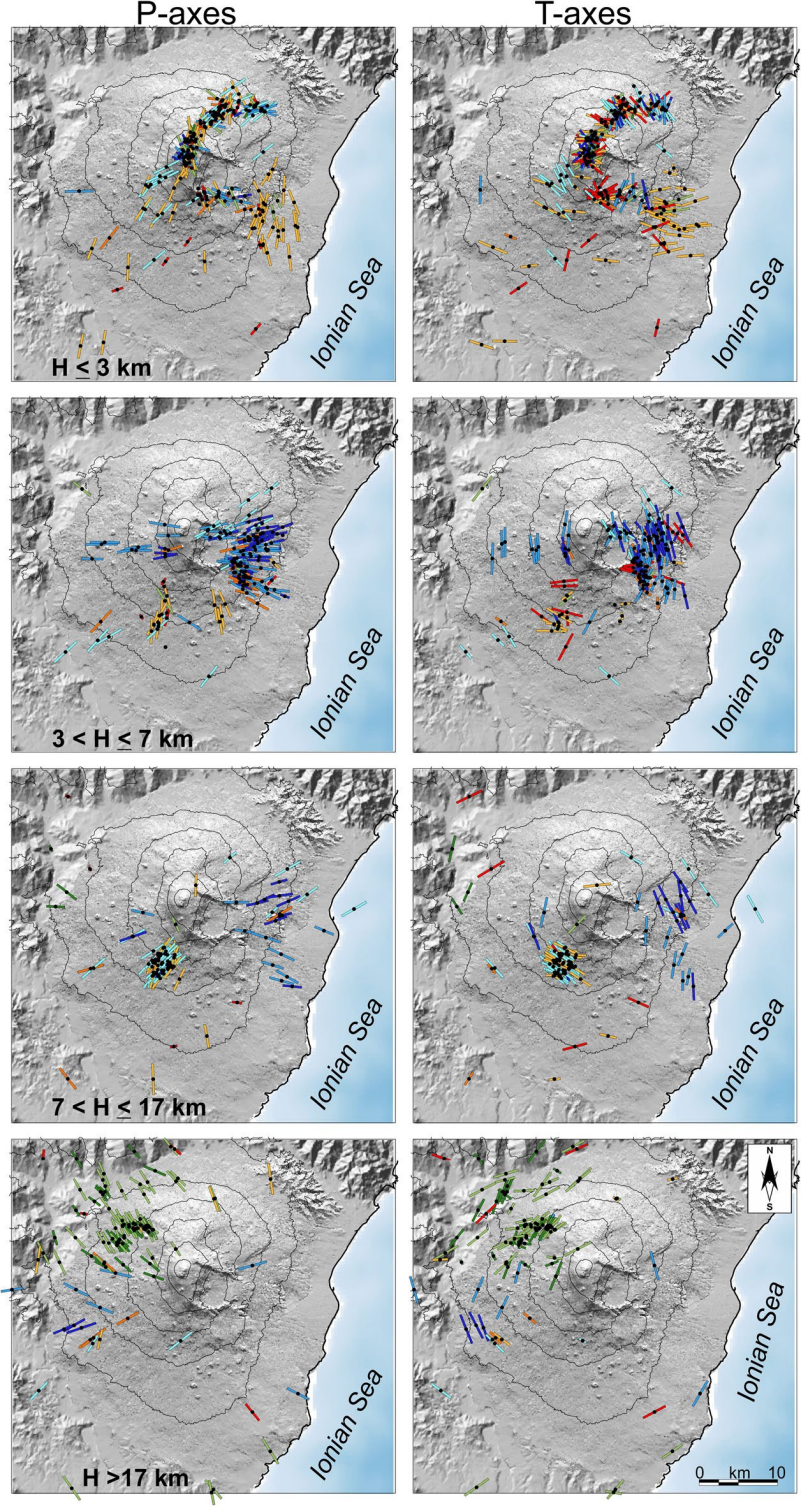
P- and T-axes of the considered focal mechanisms, at different depth levels. Colouring is the same as in Fig. 5. Topography is from Ref.^60^. The maps were created using QGIS Desktop (version 3.16.1; https://www.qgis.org/it/site/) and Generic Mapping Tools (version 6.0; https://www.generic-mapping-tools.org/).

For the interpretation of the seismo-tectonic stress field, we plotted the orientation of the P- and T-axes of each focal mechanism, distinguishing the four main depth ranges mentioned above (see Fig. 7). For the shallow group we find strongly varying directions of P-axes, in particular in the summit area. For many of these events, however, the fault plane solutions correspond to normal faulting mechanisms with almost vertical P-axes. The horizontal orientation of the T-axes reveals a more stable picture for these events. The pattern of the T-axes is consistent with a SE and ESE movement of the SE flank of the volcano.

The events populating the layer in the depth range 3–7 km are found mainly in the eastern and southern part of the area. Again, we note a marked variation of the P-axes, with a prevalent strike direction in E and ESE in the eastern part, S in the southern part and again E in the western part of the area. On the whole, the directions of the P-axes are oriented in a more or less radial geometry, pointing to a centre close to the summit area of the volcano. The T-axes make up concentric rings, again around a centre close to the summit.

In the layer between 7 and 17 km depth, the events are concentrated in the eastern and south-western quadrants. The orientation of both P- and T-axes confirm the picture outlined above, i.e. P-axes pointing to a centre close to the summit, and T-axes delineating concentric rings around this centre.

Events deeper than 17 km are found mainly in the north-western sector of the area, with P-axes striking NW. Some events located in the western side have P-axes striking E and NE. On the whole, the picture created by the orientation of the axes matches the ones found at 3–7 km and 7–17 km. At the same time, the orientations found for the events located in the NW sectors coincide with the regional stress field encountered for the central and northern part of Sicily^52,57^.

## Discussion and conclusions

Seismic and ground deformation high quality data collected during the last 20 years (2002–2021) allow developing a comprehensive geodynamical picture of Mt. Etna and the adjacent area.

Ground deformation resulting from measurements on the western flank of the volcano, displaying inflation-deflation phases, marks the eruptive activity well. In particular, besides an abrupt step noted at the beginning of the 2002/03 eruption due to the dike-forming intrusion, the curve shows a rather smooth inflation, interrupted by short time intervals of deflation. The inflation underwent some acceleration from 2015 until the beginning of 2021, after which, a period characterized by intense lava fountain activity corresponds to a marked deflation^27^.

The geographical distribution of the hypocenters reveals a complex and heterogeneous picture. On the whole, we may note a tendency of increasing focal depth from SE to NW. From the distribution of the foci we can infer four main seismic layers (i) shallow depth < 3 km below sea level, (ii) depth 3–7 km, (iii) depth 7–17 km and (iv) depth > 17 km; in addition, we identified 11 key volumes where the earthquakes are concentrated.

Characteristic of the seismicity on Mt. Etna is the presence of frequent shallow foci that can be linked to the dynamics of the volcano and the consequent instability of its eastern and southeastern flanks. Indeed, swarms repeatedly occur during and shortly before or after major eruptions, as evidenced by the major steps in the strain release curves of the shallower volumes. These jumps are related to the flank eruptions of 2002/03, 2008 and 2018 (see the strain release curves V2 and V3 in Fig. 4). Although the relationship between seismicity, ground deformation and volcanism for the surface volumes is rather ‘straightforward’, this link in the deeper layers is less clear. An interesting picture arises by analyzing the focal mechanism clusters and plotting the orientation of the P- and T-axes. As for strain release curves, the axes of the shallow events mirror the movement of the instable flank towards the E and SE. The dispersion of strike of the axes is an effect of the complex stress field caused by the magma dynamics in the shallower portion of the plumbing system acting on the faults of the volcano. On the other hand, P- and T- axes of deeper events (radial and concentric with regard to the Etna summit, respectively) clearly indicate the existence of a pressure source in the central part of the volcano. Indeed, the pressurization of the plumbing system at 3–7 km depth has been claimed by several authors (e.g. Refs.^30,49,58^) as the main source of Etna volcano inflation. This hypothesis matches well both with the orientation of the compressional and tensional axes, and with the earthquake concentration and the related strain release in that depth level. Note that the strain release curve V6 related to the events located in the eastern flank of the volcano shows the closest similarity, nearly concurrent, to the curve reporting the ground deformation. Thus, the strong seismic activity of the eastern sector can be explained by the presence of a complex system of faults that divides the eastern and southern flanks in several blocks^17^; these are destabilized under the action of increased pressure of the plumbing system. This is mirrored in a strong coupling between seismicity and deformation.

Concerning the deepest levels, some studies (e.g. Ref.^58^) claim a regional tectonic stress field, with NW–SE to N–S orientated compression being dominant at depths > 10 km. Conversely, we found that the orientation of both P- and T-axes at depths > 7 km down to 17–20 km confirms the picture outlined above, i.e. P-axes pointing to a centre close to the summit, and T-axes delineating concentric rings around this centre. In the NW sector, the P-axes of the fault plane solutions, at least as concerns events located at depths greater than 17 km, strike NW; in other words, there the direction of compression relative to the volcano and the regional one coincide.

**Figure 8:**
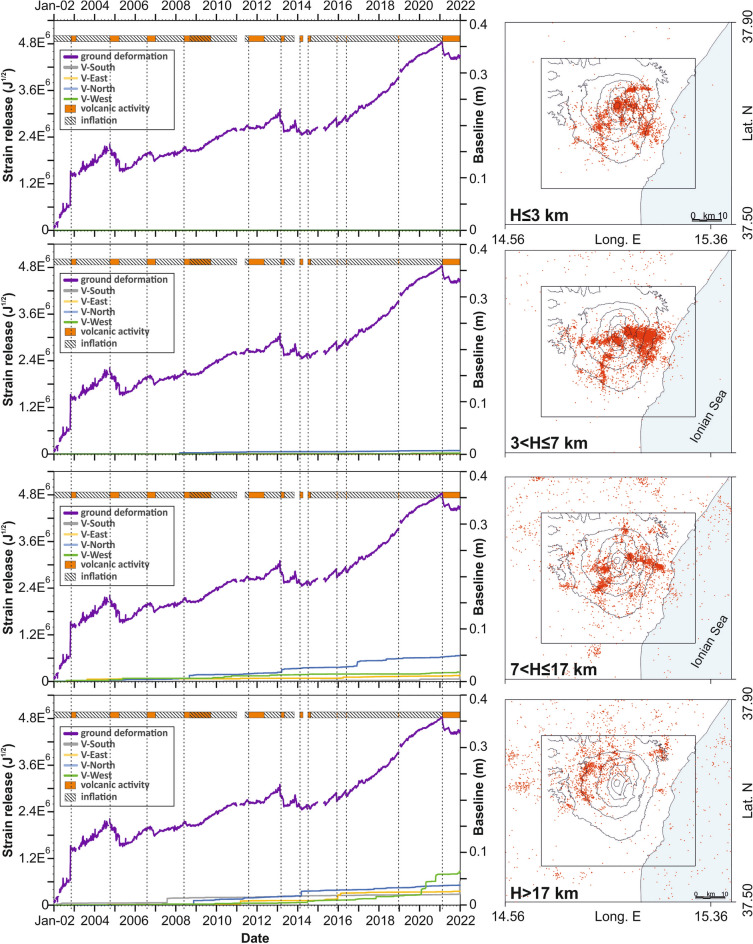
Curves of the cumulative seismic strain release, calculated within the crustal volumes of the areas adjacent to Mt. Etna (to the S, E, N and W), and their comparison with the ground deformation curve (purple). Seismic events (red dots) and the related strain release curves are separated in depth levels, indicated on the bottom left of the maps. Time intervals with enhanced volcanic activity are represented at the top by orange bars; cross-hatched bars indicate periods of inflation.

With the large time span considered here – 20 years—we obtain a reasonable coverage of all relevant sectors around the volcano, allowing to draw a comprehensive picture of stress distribution inferred from strain release curves and focal mechanism. The results indicate that the volcano related stress field dominates in the whole area of interest, both in the shallow levels as well as at greater depth. The prevalence of stress loading caused by the volcano dynamics is supported by comparing strain release curves calculated in the aforementioned 11 key volumes to those obtained for adjacent volumes (see Fig. 8). In general, the strain release in the adjacent elements is five to ten times smaller than in the volumes identified in the area of Mt. Etna. This holds even for the deepest layers, i.e. 7–17 km and > 17 km, which means that even these deep levels are affected by the processes related to the dynamics of magma movement rather than being the expression of tectonic processes acting on a regional scale. Even though we have no direct evidence, such as volcanic products erupted from that depth, some observations regarding gas emission, in particular the emission of CO_2_, SO_2_ as well as the isotope ratio He^3^/He^4^ provide clues to the activation of the deeper parts of the magma feeding system^59^ from 2015 to the end of 2018.

**Figure 9:**
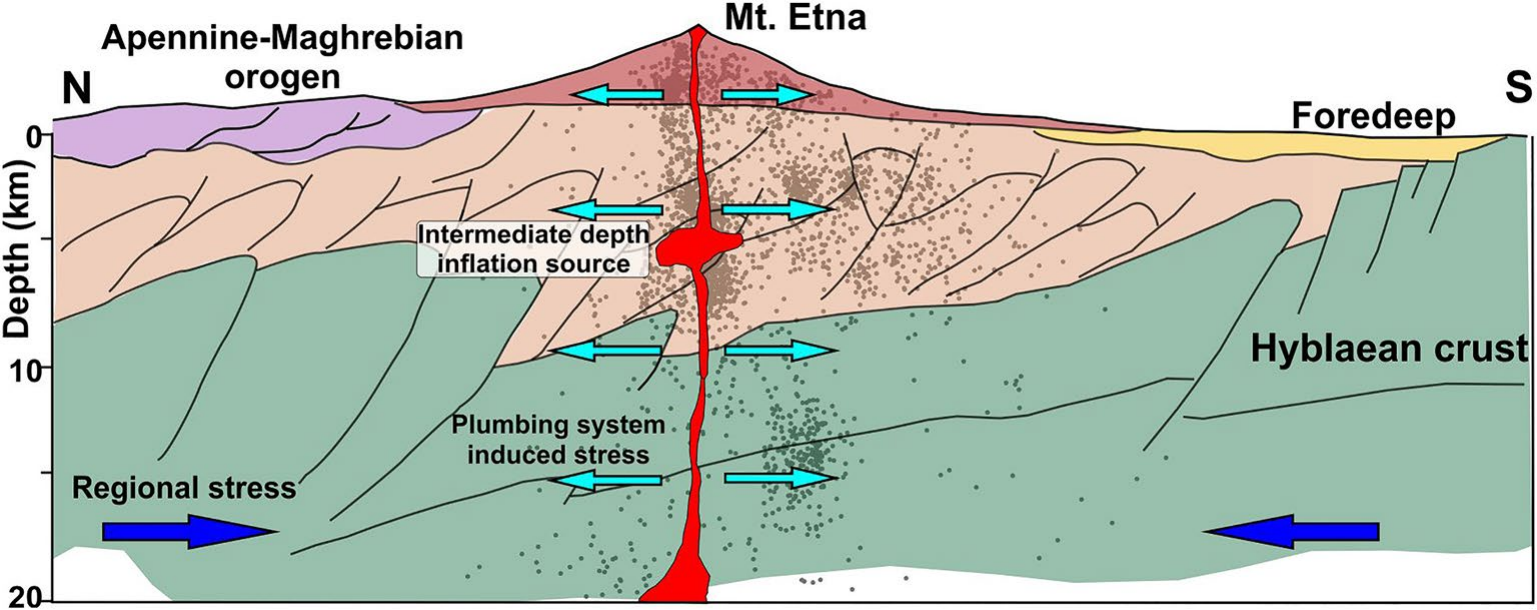
A model for stress and strain inferred from ground deformation data and seismo-tectonic analysis. The regional stress field interferes with loading caused by the dynamics of the volcano. This loading mirrors a radial compression caused by the plumbing system at the centre of the volcano and dominates the stress field encountered in the vicinity of Mt Etna.

In conclusion, the seismogenesis at Mt. Etna is closely related to the stress field generated by the magma dynamics. This is particularly evident for earthquakes occurring at shallow and intermediate levels; however, data clearly indicate that even the seismicity located at deeper depths represents the seismic response to the stress due to the interaction between regional tectonics and magmatic processes, the latter being the prevailing factor (see Fig. 9).

### Supplementary Information

Supplementary Information.

## Data Availability

The parameters (location and origin time) of the earthquakes analysed in this study are available by the *figshare* repository at 10.6084/m9.figshare.23715156.v1. The datasets of the focal mechanisms (10.13127/etnasc/efmd_2002_2021) and the time-series (2002–2021) of the distance between GPS benchmarks (10.13127/etna/eddv_2002_2021) calculated in this study are available through the Open Data Portal of the INGV.
