# Supplementary material for: Observing Etna volcano dynamics through seismic and deformation patterns
Source: Sci Rep. 2023 Aug 10;13:12951. doi: 10.1038/s41598-023-39639-9 (PMC10415380; doi:10.1038/s41598-023-39639-9)
Supplement: Supplementary file 1 — Supplementary Information. [file 41598_2023_39639_MOESM1_ESM.pdf]

Observing Etna Volcano Dynamics through Seismic and Deformation Patterns

Scarfì L.*, Aloisi M., Barberi G., Langer H.

Istituto Nazionale di Geofisica e Vulcanologia – Osservatorio Etneo, Catania, Italy

Corresponding author: luciano.scarfi@ingv.it

Introduction

This supplementary material provides information on the techniques and analyses used in the main article.

GNSS and seismic networks

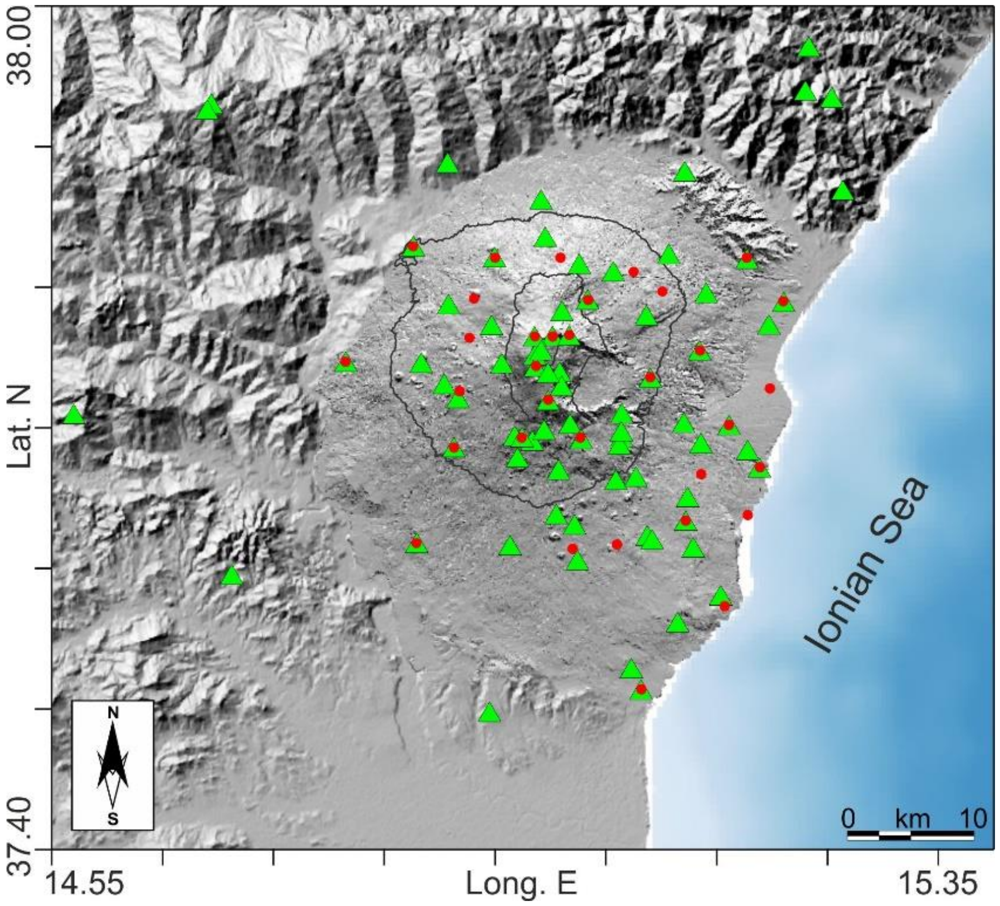

Figure S1. GPS (red circles) and seismic stations (green triangles) deployed on the Etna volcano. Topography is from[1]. The maps were created using QGIS Desktop (version 3.16.1; https://www.qgis.org/it/site/) and Surfer (version 21.1.158; https://www.goldensoftware.com/products/surfer).

Seismogenic volumes at Etna

For the definition of key volumes, we analysed the distribution of the earthquakes and seismic energy by depth (Figs S2 and S3; see the main text for further details).

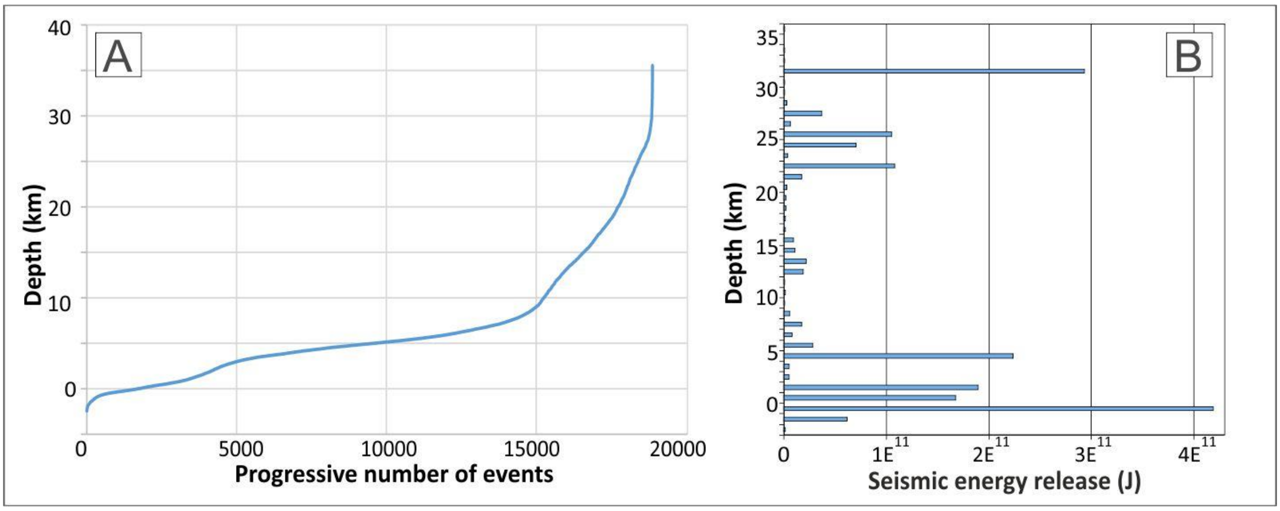

Figure S2. (A) Distribution of the earthquakes by depth. (B) Seismic energy release at various depth levels.

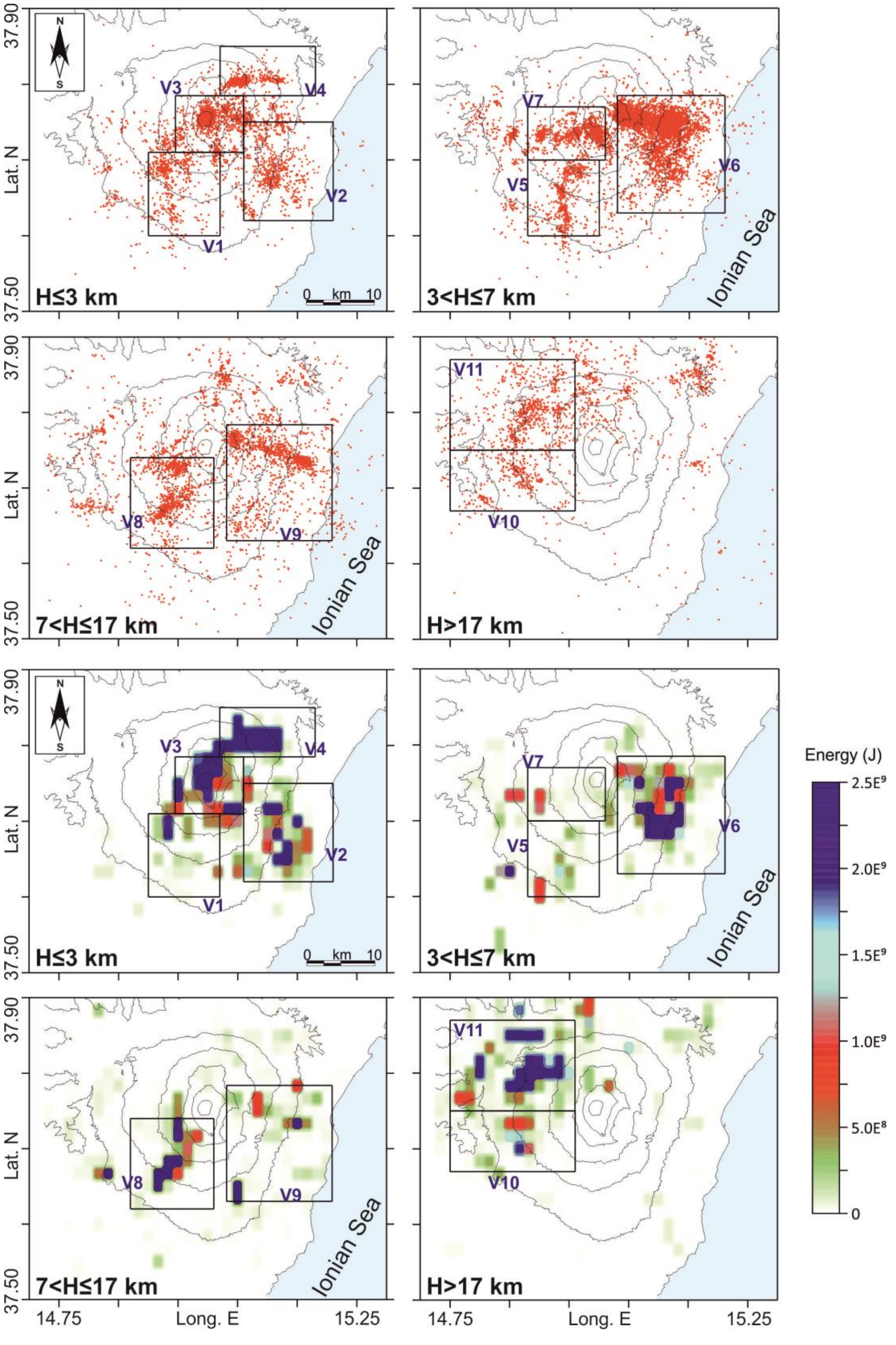

Figure S3. Distribution of earthquakes (red dots; top) and related energy release (bottom) at different depth levels during the period 2002-2021. The boxes (V1-V11) indicate the key volumes identified in the study.

Table 1. Number of seismic events within the 11 key volumes.

Volumes	V1	V2	V3	V4	V5	V6	V7	V8	V9	V10	V11
N. events	672	940	3,100	754	523	4,977	884	1,080	1,843	398	659

Measure of similarity between curves

(Dis)similarity of curves can be expressed considering the areas under the curves (AUC). In figure S4, the hatched areas represent the part which is covered only by one curve. It represents the sum of the absolute differences between the two curves. We can calculate the ratio of the hatched areas with reference to the area under one curve - for instance the blue one. In the example (a), the two curves differ strongly since the red curve has large values. The hatched area is over 50% with reference to the blue AUC. (b) and (c) represent cases where the hatched part is relatively low since the differences between the red and blue curves are minor. (d) is an example where the hatched part is again over 50% with reference to the AUC of the blue reference curve.

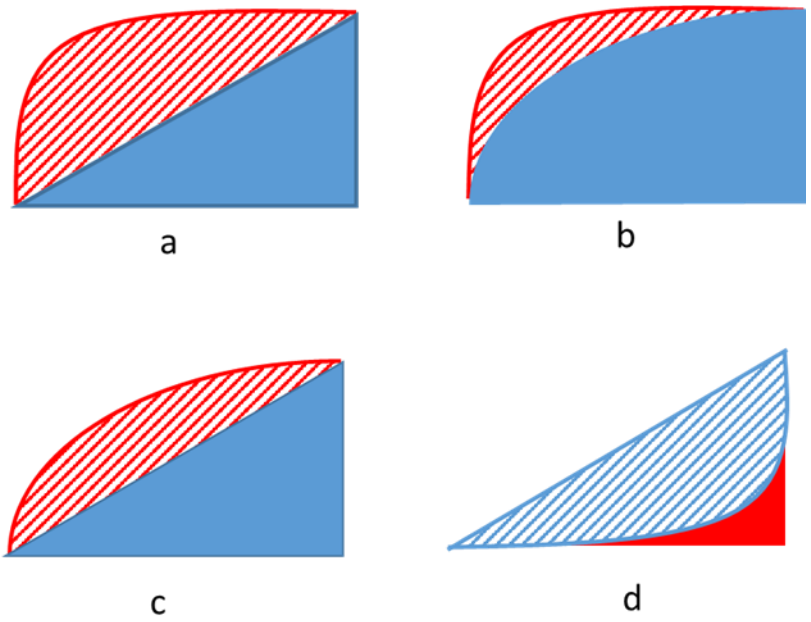

Figure S4. Similarity of curves expressed considering the areas under the curves. The hatched areas represent the part which is covered only by one curve (see text).

Unsupervised learning

In unsupervised learning we ask the computer to identify groups or clusters of similar patterns on the basis of a measure of (dis)similarity. Here we focus on techniques which identify prototypes representing a number of patterns making part of a cluster. The methods allow a considerable data reduction and simplification of interpretation. As the patterns of a cluster are considered to have similar characteristics, we do not lose much information by neglecting the internal variability among the patterns of the same cluster.

Self-Organizing Maps (SOM)

Following a strategy proposed by[2], we used an unsupervised pattern recognition technique to discover underlying hidden structures in the focal mechanism dataset. In particular, we utilised an updated version of the software KKAnalysis[3, 4], which offers different types of unsupervised classifiers. As a first step, the dataset was processed by applying so-called Kohonen Maps, also known as 'Self Organizing Maps' (SOM).

The design of SOM follows two key ideas: reducing the number of objects, i.e. data compaction and reducing the dimensionality of the problem. The first goal is achieved by identifying prototypes of patterns, each of which represents a number of samples with a reasonable degree of similarity. These

prototypes are essentially small clusters, referred to as ‘nodes’ in the SOM jargon. A further characteristic of the SOM regards the reduction of dimensionality. In particular, the SOM projects a multidimensional input space on a two-dimensional grid (Kohonen maps), on which various homogeneous areas can be identified (Fig. S5).

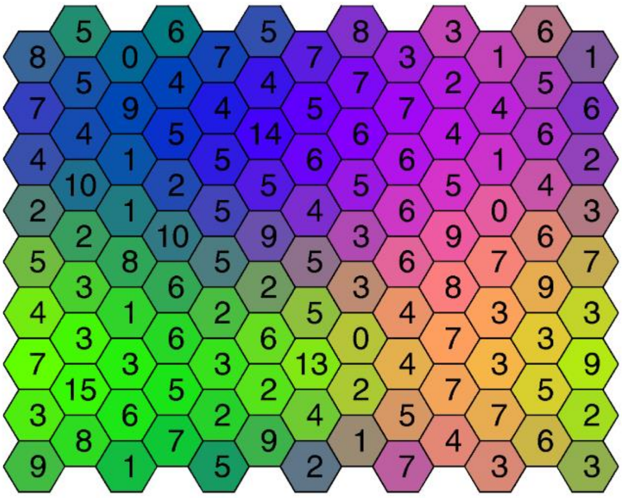

Figure S5. Distribution of nodes deriving from the SOM analysis, projected into a 2D map. Numbers indicate the elements clustered in each node. Nodes placed on opposite margins may represent patterns close to each other and have similar colours.

Training of a SOM

At the beginning of the training each node of the SOM is assigned an initial weight vector $\boldsymbol{W}_i$, e.g., by assigning random values. The $\boldsymbol{W}_i$ have the same dimensionality as the original feature vectors. The training of the SOM consists of an iterative procedure, considering the differences (here Euclidean)

$$D_{ij} = \sqrt{(\boldsymbol{W}_i - \boldsymbol{V}_j)^T (\boldsymbol{W}_i - \boldsymbol{V}_j)}$$

between the normalised input vector $\boldsymbol{V}_j$ and the weights $\boldsymbol{W}_i$ of the nodes. The identification of the closest node to the actual input vector, i.e., the best matching unit (BMU) for the j-th pattern is a key step. Besides updating the weights of the BMUs, neighbouring nodes lying within a certain radius of influence are considered as well. This is an important characteristic of SOM training, as it makes sure that weights of nodes situated closely to each other on the sheet will be updated in a similar way[*]. Once BMU and nodes falling within the area of influence have been identified, their weights are gradually adjusted according to the so-called learning rate. The rate to which the weight of the nodes is adjusted decreases with the distance Δ between each node and the BMU. The upgrade of weights follows the relationship:

$$\boldsymbol{W}_i(t+1) = \boldsymbol{W}_i(t) + \varphi(\Delta, t) \cdot \lambda(t) \cdot D_{ij}(t)$$

where φ describes the distance dependence of the upgrade of a node. $\varphi(\Delta,t)$ can have various shapes, such as Gaussian, inverse parabolic, boxcar (see[3, 4]). φ has its maximum for the BMU, nodes outside the radius of influence are not upgraded at all. During a cycle of training, this procedure is repeated for all input vectors. The stabilisation of the map is achieved by both learning rate and radius of influence at any step of iteration. Eventually, the map will depict the input vectors, the BMU, and the weight vectors calculated.

[*] Ideally, at the end of the training process, we obtain a SOM with ‘topological fidelity’. Nodes situated closely to each other on the map are also close to each other in the original feature space.

Visualization of SOM results

Visualization becomes very effective when the characteristics of patterns are represented using color codes defined in a “RGB” (red-green-blue) system. In this strategy we exploit the 2D principal component analysis (PCA) carried out on the covariance matrix of the weight vectors, considering the values of a feature vector in a system spanned by the two principal components. In our choice, the saturation of the ‘red’ component depends on the value of the first principal component, ‘green’ on the value of the second one, ‘blue’ is obtained by the subtraction 1-‘green' value. In this way, colour codes of the nodes depend on the node’s position on the map. Nodes which are close to each other on the map represent similar characteristics and are assigned a similar colour code (Fig. S5). The colour code of the nodes provides a synopsis of classification results as they allow an immediate and intuitive comparison of the groups. For instance, they can be applied in the analysis of spatial distributions of patterns (Fig. S6).

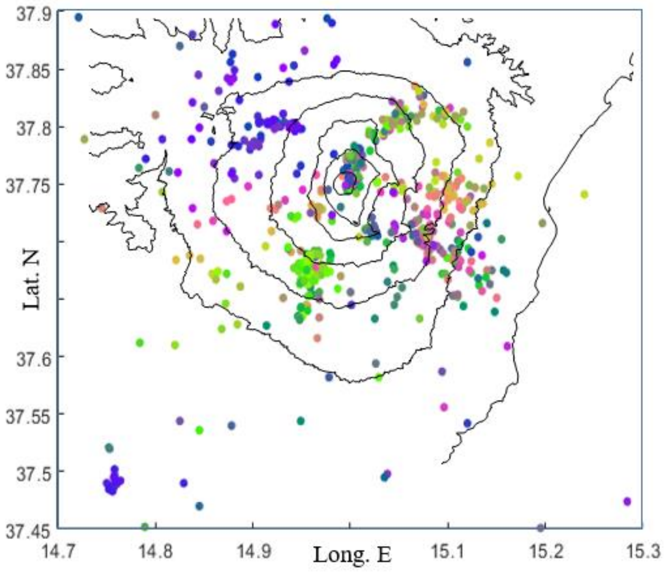

Figure S6. Distribution of the epicentres of the focal mechanisms used in the study, coloured according to the SOM node membership (see Fig. S5).

Clustering

Clustering is carried out to a reduce a large numbers of patterns to a few prototypes. The measure of the heterogeneity of the *k*-th cluster is expressed as the sum of the squared distances of the patterns in each cluster from their centroid vector. In Partitioning Clustering, we have to fix the number of clusters a priori. The ‘K-means’ algorithm is perhaps the most popular clustering method for its straightforward concept and computational simplicity.

Consider the dispersion S in our ensemble consisting of n feature vectors $\boldsymbol{x}_i$ having the global mean $\bar{\boldsymbol{x}}$

$$S = \sum_{i=1}^{n} ||\boldsymbol{x}_i - \bar{\boldsymbol{x}}\ ||^2; \boldsymbol{S}_{Tot} = \sum_{i=1}^{n} (\boldsymbol{x}_i - \bar{\boldsymbol{x}})(\boldsymbol{x}_i - \bar{\boldsymbol{x}})^T$$

denoting by $\boldsymbol{S}_{Tot}$ the dispersion matrix of the whole data set. Having a partition such that each $\boldsymbol{x}_i$ belongs to some cluster j, we measure the dispersion within each cluster by

$$\boldsymbol{S}_j = \sum_{i=1}^{m} (\boldsymbol{x}_i - \bar{\boldsymbol{x}}_j)(\boldsymbol{x}_i - \bar{\boldsymbol{x}}_j)^T$$

The dispersion between the k clusters is

$$\boldsymbol{S}_C = \sum_{j=1}^{k} m_j (\bar{\boldsymbol{x}}_j - \bar{\boldsymbol{x}}\ )(\boldsymbol{x}_j - \bar{\boldsymbol{x}})^T$$

where we assume that each cluster is composed of m_j samples. It can be shown that

$$\boldsymbol{S}_{Tot} = \sum_{j=1}^{k} \boldsymbol{S}_j + \boldsymbol{S}_C$$

i.e., the total dispersion is given by the sum of the dispersions measured within the clusters and the dispersion measured between the cluster centroids $\bar{\boldsymbol{x}}_j$.
In crisp K-means clustering we can use the "Davis-Bouldin" Index[5] for choosing the number of clusters. This is obtained by comparing the average similarity encountered among all clusters to the largest one. The similarity between clusters *i* and *j* is given by

$$R_{ij} = (s_i + s_j) / \| \mathbf{c}_i - \mathbf{c}_j \|$$

where s_i and s_j are the variance measures in each cluster; $\mathbf{c}_i$ and $\mathbf{c}_j$ are the corresponding cluster centroid vectors. With $R_i = \max(R_{ij})$, the DBI is obtained from the average of the R_i taken over all k clusters, i.e.,

$$\mathrm{DBI} = 1/k \,\Sigma_i R_i$$

Prototypes of the moment tensor clusters

Cluster prototypes can be obtained as the centroids of the moment tensors belonging to a certain cluster. However, averages may fail to mirror the characteristics of the single samples as the sum of a number of double couple moment tensor does not necessarily still represent a pure double couple mechanism, but may include other components, such as CLVD or isotropic components. An alternative of identifying prototypes exploits the so-called Kagan angle[6], which is determined from the orientation of the principal axes P, T, and B.
The orientation of axes is given by the orientation matrix

$$\boldsymbol{D} = \begin{vmatrix} t_1 & p_1 & b_1 \\ t_2 & p_2 & b_2 \\ t_3 & p_3 & b_3 \end{vmatrix}$$

where each vector $\boldsymbol{t}$, $\boldsymbol{p}$, $\boldsymbol{b}$ is given by plunge and azimuth angles. For example

$$t_1 = \cos(\alpha_t)\cos(\beta_t)$$
$$t_2 = \cos(\alpha_t)\sin(\beta_t)$$
$$t_3 = \sin(\alpha_t)$$

(see Kagan, 2007). The rotation angle is obtained from

$$\Phi = \arccos[1/2(|\boldsymbol{t}\cdot\boldsymbol{t}'| + |\boldsymbol{p}\cdot\boldsymbol{p}'| + |\boldsymbol{b}\cdot\boldsymbol{b}'| - 1)]$$

for $\Phi \leq 90°$ and

$$\Phi = \arccos[1/2(\boldsymbol{t}\cdot\boldsymbol{t}' + \boldsymbol{p}\cdot\boldsymbol{p}' + \boldsymbol{b}\cdot\boldsymbol{b}' - 1)]$$

for $\Phi > 90°$.
The angle Φ is known as the 'Kagan' angle. In theory, up to four solutions may be obtained, in practice the minimum angle is used. It can be shown that the minimum Kagan angle is always $\leq 120°$.
A code for the computation of the Kagan angles has been made available by[7].

References

1. Ryan, W. B. F. et al. Global Multi-Resolution Topography (GMRT) synthesis data set. Geochem. Geophys. Geosyst. 10(Q03014), https://doi.org/10.1029/2008GC002332 (2009).
2. Scarfì, L., Langer, H., Messina, A. & Musumeci, C. Tectonic regimes inferred from clustering of focal mechanisms and their distribution in space: application to the Central Mediterranean Area. *J. Geophys. Res. Solid Earth* **126**, e2020JB020519, https://doi.org/10.1029/2020JB020519 (2021).
3. Messina, A. & Langer, H. Pattern Recognition of volcanic tremor data on Mt. Etna with KKAnalysis – a software program for unsupervised classification. *Computer and Geosciences* **37** (7), 953-961, https://doi.org/10.1016/j.cageo.2011.03.015 (2011).
4. Langer, H., Falsaperla, S. & Hammer, C. Advantages and Pitfalls of Pattern Recognition: Selected Cases in Geophysics. In *Computational Geophysics*, **3**, (ed. Elsevier, 2020).
5. Davies, D. L. & Bouldin, D. W. A cluster separation measure. *IEEE Transactions on Pattern Recognition and Machine Learning* **1** (2), 224-227, (1979).
6. Kagan, Y.Y. Simplified algorithm for calculating double-couple rotation. *Geophys. J. Int.* **171** (1), 411-418, https://doi.org/10.1111/j.1365-246X.2007.03538.x (2007).
7. Petr Kolar. Kagan angle (https://www.mathworks.com/matlabcentral/fileexchange/70040-kagan-angle), MATLAB Central File Exchange, 2022.
